# Suppressive Effects of Rosmarinic Acid Rich Fraction from Perilla on Oxidative Stress, Inflammation and Metastasis Ability in A549 Cells Exposed to PM via C-Jun, P-65-Nf-Κb and Akt Signaling Pathways

**DOI:** 10.3390/biom11081090

**Published:** 2021-07-23

**Authors:** Komsak Pintha, Wittaya Chaiwangyen, Supachai Yodkeeree, Maitree Suttajit, Payungsak Tantipaiboonwong

**Affiliations:** 1 Division of Biochemistry, School of Medical Sciences, University of Phayao, Phayao 56000, Thailand; komsakjo@gmail.com (K.P.); wittaya.ch@up.ac.th (W.C.); maitree.suttajit@gmail.com (M.S.); 2 Department of Biochemistry, Faculty of Medicine, Chiang Mai University, Chiang Mai 50200, Thailand; yodkeelee@hotmail.com

**Keywords:** particulate matter, reactive oxygen species, inflammation, metastasis, perilla, rosmarinic acid rich fraction

## Abstract

Particulate matter from forest fires (PMFF) is an environmental pollutant causing oxidative stress, inflammation, and cancer cell metastasis due to the presence of polycyclic aromatic hydrocarbons (PAHs). Perilla seed meal contains high levels of polyphenols, including rosmarinic acid (RA). The aim of this study is to determine the anti-oxidative stress, anti-inflammation, and anti-metastasis actions of rosmarinic acid rich fraction (RA-RF) from perilla seed meal and its underlying molecular mechanisms in A549 cells exposed to PMFF. PMFF samples were collected via the air sampler at the University of Phayao, Thailand, and their PAH content were analyzed using GC-MS. Fifteen PAH compounds were detected in PMFF. The PMFF significantly induced intracellular reactive oxygen species (ROS) production, the mRNA expression of pro-inflammatory cytokines, MMP-9 activity, invasion, migration, the overexpression of c-Jun and p-65-NF-κB, and Akt phosphorylation. Additionally, the RA-RF significantly reduced ROS production, IL-6, IL-8, TNF-α, and COX-2. RA-RF could also suppress MMP-9 activity, migration, invasion, and the phosphorylation activity of c-Jun, p-65-NF-κB, and Akt. Our findings revealed that RA-RF has antioxidant, anti-inflammatory, and anti-metastasis properties via c-Jun, p-65-NF-κB, and Akt signaling pathways. RA-RF may be further developed as an inhalation agent for the prevention of lung inflammation and cancer metastasis induced by PM exposure.

## 1. Introduction

In the northern region of Thailand, air pollution is an annual and severe environmental issue. Long-term exposure to air pollution causes both acute and chronic health effects [1]. The burning of the forests and agricultural wastes are associated with smoke pollution in this region [2]. Fine particulate matter ≤10 μm (PM10, PM2.5) has been identified as one of the most prevalent human health risks due to its deep infiltration into the respiratory tract [3,4]. These particles have the capacity to accumulate various toxic components, including polycyclic aromatic hydrocarbons (PAHs) [5]. PAHs carry potential mutagens and carcinogens that can promote inflammation and tumor progression [6,7]. PM can activate free radical species generation, which strongly influences the development of oxidative DNA damage, eventually causing DNA mutation [6].

Unbalanced reactive oxygen species (ROS) production and antioxidant activity increases oxidative stress that can trigger a cascade of inflammation signaling pathways and cancer cell metastasis [8,9]. PM-induced oxidative stress has been accepted as a key molecular mechanism of many inflammatory modulators, such as tumor necrosis factor-α (TNF-α), interleukins IL-1, IL-6, IL-8, and COX-2, including MMP-9 expression and cancer cell metastasis [10,11,12,13]. The elevation of inflammatory mediators and the MMP-9 degradation enzyme are associated with Akt, NF-KB, and AP1 signaling pathways [14].

Previous studies have demonstrated that polyphenol extract from medicinal plants suppressed ROS production, proinflammatory cytokine expression, and MMP degradation enzymes in PM-treated cells [15,16]. *Perilla frutescens* L. (Nga-mon in Thai), belonging to the mint family, has been widely used for consumption and as a medicinal herb in Asian countries including Thailand, Japan, Korea, China, and India [17]. Chemically, the perilla seed contains high levels of essential fatty acids, mainly α-linolenic acid (~60%), linoleic acid (~20%) [18], and phenolic compounds. Among these phenolic acids, rosmarinic acid (RA) is reportedly predominant in the ethanolic extracts of perilla [19,20,21,22]. The RA content in 29 species of Labiatae family using water:methanol:2-propanolwas extract in the range 0.0 ± 0.00 to 58.5 ± 1.4 mg/g [23] and Lamiaceae plants (*Ocimum sanctum*, *O. basilicum*, *O. americanum* and *Metha cordifolia* opiz.) in the range 1.16 to 8.45 mg/g [24]. In 2020, Sik et al. found that the RA amount in Lamiaceae herbs (lemon balm, peppermint, oregano, rosemary, sage, and thyme) by various extraction techniques was in the range 7.4 ± 0.2 to 40.1 ± 1.0 mg/g [25]. Our previous report found that the 70% ethanolic extracts and enriched fractions from perilla leaves, seeds, and seed meal contained the highest content of RA in the approximate range (80–158 mg/g extract) and exerted antioxidant, anti-inflammatory, and anti-cancer effects [19,20,21,22]. To our knowledge, however, the effects of RA-RF on the induction of PMFF causing oxidative stress, inflammation and metastasis in A549 cells have never been investigated or published. 

In this study, we aimed to explore the effects of RA-RF on ROS production, the mRNA expression of inflammatory mediators (TNF-α, IL-6, IL-8, COX2 enzyme), and invasion, migration, and MMP-9 activity in PMFF-induced human lung epithelial A549 cells. Furthermore, the molecular mechanisms of Akt, p65-NF-κB, and c-Jun signaling pathways leading to the reduction of anti-oxidative stress, inflammatory cytokines, and cancer cell metastasis were also investigated.

## 2. Materials and Methods

### 2.1. PMFF Sampling and GC-MS Analysis

PMFF samples were collected using the nitrocellulose membrane filter (Toyo Roshi Kaisha, Ltd., Tokyo, Japan) at the University of Phayao located in the northern region of Thailand. The sampling area was near a forest fire incident. After sampling, PAH compounds including acenaphthene, acenaphthylene, anthracene, benz[a]anthracene, benzo[a]pyrene, benzo[b]fluoranthene, benzo[ghi]perylene, benzo[k]fluoranthene, chrysene, dibenz[a,h]anthracene, fluoranthene, fluorene, indeno[1,2,3-cd]pyrene, naphthalene, phenanthrene, and pyrene were determined using an Agilent 7820A gas chromatograph coupled to an Agilent 5977E [26].

### 2.2. Rosmarinic Acid Rich Fraction (RA-RF) Preparation

Green Perilla (Perilla frutescens var. acuta) were grown between August 2018–January 2019 from Phayao province (longitude 100°3′3.564″, latitude 19°12′9.36″, altitude 740 m). Perilla seeds were harvested in February, and a voucher specimen (Code: QBG93756) of the plant was provided and preserved at the Queen Sirikit Botanic Garden Herbarium, Chiang Mai, Thailand. Rosmarinic acid rich fraction (RA-RF) was prepared via the following method [20]. The seed meal was extracted in 70% ethanol and the dried extract was subsequently fractionated by hexane, dichloromethane, and ethyl acetate. All fractions were assessed for the level of RA content using ultra-high-pressure liquid chromatography, UHPLC (Agilent Technologies, Inc., Santa Clara, CA, USA).

### 2.3. Cell Culture

Human lung epithelial cells (A549) were purchased from the American Type Culture Collection (ATCC^®^ CCL-185™). The cells were maintained in Opti-MEM medium (Gibco, cat#31985062) supplemented with 100 U/mL penicillin-streptomycin (Gibco, cat#15140) and 10% fetal bovine serum (FBS; *v*/*v*) (Gibco, cat#10270) and incubated at 37 °C in a humidified incubator containing 5% CO_2_.

### 2.4. Cell Viability Assay

Cell viability was performed using the MTT assay [22]. Different concentrations (0–200 µg/mL) of RA-RF or PMFF were incubated with the A549 cells in each well of 96-well plate for 24 h. For the co-treated assay, the cells were incubated with RA-RF (0–200 µg/mL) for 2 h. Then, all the samples were exposed to 200 µg/mL of PMFF. After 24 h of exposure, 15 µL of MTT solution was added and further incubated for 4 h followed by 200 µL of dimethyl sulfoxide (DMSO) until the formazan crystals dissolved in the cells. The optical density of the samples was measured at the wavelengths of 570 and 630 nm using a microplate reader. The data were independently performed in triplicate.

### 2.5. Cell Apoptosis Assay

The Muse™ Annexin V and Dead Cell Kit (Merck Millipore, Guyan-court, France) was used for the determination of A549 cell apoptosis. The cells were collected, washed with phosphate buffer saline (PBS), resuspended in an Opti-MEM medium with the Kit reagent, and then incubated for 20 min at room temperature in the dark. A Muse™ Cell Analyzer was used to measure the data [27].

### 2.6. Reactive Oxygen Species (ROS) Measurement

The intracellular ROS production was analyzed via 2′-7′-Dichlorodihydrofluorescein diacetate (DCFH-DA) procedure. Briefly, the A549 cells were grown in a 96-well plate and allowed to attach overnight and then washed twice with phosphate-buffered saline (PBS; pH 7.4). The cells were incubated with the fluorescent dye DCFH-DA (20 μM) at 37 °C for 2 h. The excess DCFH-DA was removed by washing twice with PBS. The cells were co-treated with varying concentrations of RA-RF (5, 10 and 20 μg/mL) and PMFF (200 μg/mL) for another 30 min at 37 °C. To analyze the intracellular ROS production, the fluorescence intensity of oxidized dichlorofluorescein (DCF) was measured at 485 nm excitation and 530 nm emission wavelengths [22]. 

### 2.7. Total RNA Extraction and cDNA Synthesis

The A549 cells were seeded at 1.2 × 10^6^ cells per well into a 6-well plate and allowed to attach overnight. The cells were pretreated with varying concentration of RA-RF (5, 10 and 20 μg/mL) and incubated at 37 °C in 5% CO_2_ incubator for 2 h. Then, 200 µg/mL of PMFF was added and incubated for 16 h. Total RNA was extracted from the cells using NucleoSpin^®^ RNA kit as recommended by the manufacturer’s instruction (Macherey-Nagel, Dueren, Germany). A total of 1 µg of total RNA was synthesized for cDNA using ReverTra Ace^®^ qPCR RT Kit according to the manufacturer’s protocol (TOYOBO, Osaka, Japan) [22].

### 2.8. Quantitative Real-Time PCR (qPCR)

The expressed profiles of pro-inflammatory cytokine genes were investigated via quantitative real-PCR (qPCR). The genes and their primer pairs are listed in Table 1. GAPDH was used as an internal control for all qPCR experiments and was amplified using a primer pair listed in Table 2. Each reaction was performed in a total volume of 20 µL using 5 µL of cDNA (dilute 1:5), and 0.8 µL of 10 µM primers of each gene-specific primer pair with 2× SensiFAST SYBR^®^ Lo-ROX (Bioline, Singapore Science Park II, Singapore). All the qPCRs were performed in the 7500 Real-time PCR system (Applied Biosystems, Foster City, CA, USA). Cycling parameters were started with initial denaturation at 95 °C for 10 min, followed by 40 cycles of 95 °C for 15 s and annealing with extension at 60 °C for 60 s [22]. The relative expression was determined via the 2^−^^ΔΔ^Ct method [28].

### 2.9. Cell Invasion and Migration Assay 

Invasion and migration were accessed using 24-well hanging cell culture inserts with 8 μm pore size (Millicell^®^, Munich, Germany). The A549 cells with the number of 1.25 × 10^5^ were incubated with different concentrations of RA-RF (0–20 µg/mL) with or without PM (200 µg/mL), and placed into the upper chamber of inserts pre-coated with Matrigel or without Matrigel for migration assay. The medium in the lower chamber contained 20% FBS for the chemoattractant and was incubated for 24 h at 37 °C in 5% CO_2_. The non-invaded cells were removed with cotton swabs, while invaded cells at the lower surface of the membrane were fixed with cold 80% ethanol and stained with 0.1% crystal violet. The stained cells were decolorized with 1% acetic acid, and the absorbance at 570 nm was measured [29].

### 2.10. Gelatin Zymography 

The A549 cells were co-treated with PMFF and RA-RF for 24 h, and the secretions were collected and analyzed by using 10% polyacrylamide gels electrophoresis containing 0.1% (*w*/*v*) gelatin in nonreducing condition. The electrophoretic gel was removed of sodium dodecyl sulfate (SDS) by washing the gel twice with 2.5% Triton X-100 for 30 min at room temperature. Then, the gel was kept for 18 h in an activating buffer (50 mM Tris-HCl, 200 mM NaCl, and 10 mM CaCl_2_, pH 7.4) at 37 °C, Coomassie Brilliant Blue R (0.1%, *w*/*v*) was used for staining the gel. De-stained solvent is 30% methanol with 10% acetic acid [30]. The clear band on the blue background shows the MMP-9.

### 2.11. Western Blotting 

A549 cells were seeded and allowed to attach overnight, and then pretreated with varying concentrations (5–20 μg/mL) of RA-RF. After incubation for 2 h, the pretreated cells were induced with 200 µg/mL of PMFF for 30 min. To collect the protein, the cells were washed with PBS buffer and then lysed using lysis buffer (all buffer supplemented with protease inhibitors, SERVA GmbH, Heidelberg, Germany), followed by three thaw-freeze cycles. The protein concentration was quantified using the Bradford method (Sigma-Aldrich, Munich, Germany). In order to detect protein expression, the proteins were separately loaded into the 10% and then transferred onto a nitrocellulose membrane (Hybond-P; GE Healthcare UK Limited, Amersham Place Little Chalfont, Buckinghamshire, UK). The membranes were initially blocked with a blocking buffer for 1 h. Subsequently, the membrane was immunoblotted with the first specific antibody diluted in PBS buffer at 4 °C overnight. After washing three times with washing buffer, the membrane was incubated with HRP-conjugated anti-rabbit IgG antibody (1:5000 dilution) for 1 h at room temperature. After intensive washing, the ECL Western blotting reagent kit (GE Healthcare) was used to detect the protein according to the manufacturer’s instructions [30]. For all Western blotting analyses, the primary monoclonal antibodies (Rabbit mAb) were purchased from Cell Signaling Technology Inc. (Danvers, MA, USA) and diluted 1:1000; phospho-c-Jun (Ser73) (D47G9) XP^®^ Rabbit mAb #3270 (48kD), phospho- p65-NF-κB (Ser536) (93H1) Rabbit mAb #3033, phospho-Akt (Ser473) Antibody #9271 and β-Actin (13E5) Rabbit mAb #4970. 

### 2.12. Statistical Analysis

The one-way ANOVA followed by Tukey’s test was performed to statically analyze all experiments using GraphPad Prism (GraphPad Software, San Diego, CA, USA). All graphs are shown as mean ± standard error of the mean (SEM) value. Significant differences were considered at *p* < 0.05.

## 3. Results

### 3.1. The PAHs Concentrations in PMFF

PMFF were collected to determine the concentration of PAHs (Table 2). The 15 PAH compounds were detected, and their concentrations ranged from 2.094 ± 0.016 (benzo[ghi] perylene) to 0.072 ± 0.004 ng/m^3^ (acenaphthene). In this study, the high molecular weight PAHs, which are well known as inflammatory agents and lung cancer carcinogens [31], were mostly found and clearly higher than PAHs of low molecular weight.

**Table 2:** The PAHs compounds of PMFF analyzed by GC-MS.

Compounds	PAHs Concentration (ng/m^3^)	Mol. wt. (g mol^−1^) [32]	Class [33]
**Low molecular weight**			
Naphthalene	0.082 ± 0.003	128	-
Acenaphthylene	0.083 ± 0.000	152	-
Acenaphthene	0.072 ± 0.004	154	-
Phenanthrene	0.208 ± 0.008	178	-
Anthracene	0.196 ± 0.011	178	-
Fluorene	ND	166	-
**High molecular weight**			
Benzo[ghi]perylene	2.094 ± 0.016	276	3
Indeno[1,2,3-cd]pyrene	1.803 ± 0.036	276	2B
Benzo[b]fluoranthene	1.531 ± 0.017	252	2B
Benzo[a]pyrene	0.764 ± 0.017	252	1
Benzo[k]fluoranthene	0.609 ± 0.031	252	2B
Dibenz[a,h]anthracene	0.509 ± 0.006	278	2A
Fluoranthene	0.487 ± 0.016	202	3
Pyrene	0.396 ± 0.004	202	3
Chrysene	0.282 ± 0.001	228	2B
Benzo[a]anthracene	0.184 ± 0.003	228	2B

“ND” = Not Determined.

### 3.2. Quantification of Rosmarinic Acid Rich Fraction (RA-RF)

Previous studies have demonstrated that rosmarinic acid (RA) functions as an anti-oxidant anti-inflammatory and anti-cancer agent [34]. Therefore, we extracted the RA-rich fraction from perilla seed meal. In order to assess the amount of RA in each fraction of the extracted process, we performed a UHPLC analysis. The RA contents in the crude ethanolic, hexane, dichloromethane, ethyl acetate, and water fraction were 70.89 ± 0.73, 54.92 ± 2.57, 29.95 ± 0.44, 600.32 ± 14.61 and 38.20 ± 1.04 mg RA/g extract, respectively. It was found that the ethyl acetate fraction contained the highest amount of RA; therefore, this RA-RF was selected for its further biological effects on PMFF-induced A549 cells.

### 3.3. RA-RF Enhances Cell Viability and Apoptosis of PMFF-Induced A549 Cells

**Figure 1:**
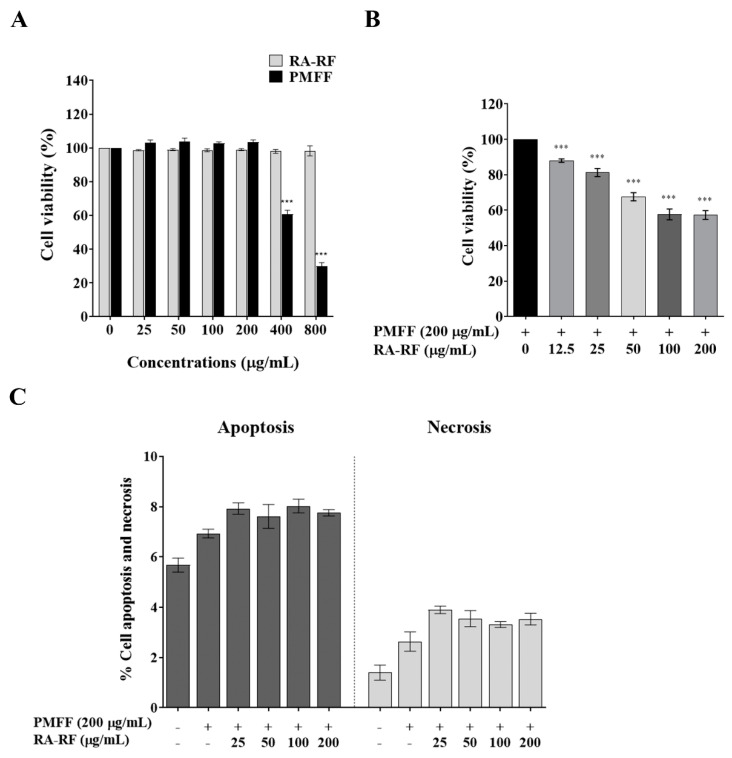
Cell viability of A549 cells treated with different concentrations (0–800 µg/mL) of the RA-RF or PMFF (**A**) and pre-treated with RA-RF (0–200 µg/mL) following by adding 200 µg/mL of the PMFF (**B**). Cell apoptosis and necrosis of A549 cells treated with PMFF (200 µg/mL) in the presence of RA-RF (0–200 µg/mL) for 24 h (**C**). The mean ± standard error of the mean (SEM); *** *p* < 0.001. The data were independently performed in triplicate.

As shown in Figure 1A, the MTT assay revealed that cell viability was observed after treating RA-RF (0–800 μg/mL). The toxic dose of PMFF was observed to be significant at 400–800 μg/mL when compared to the control. Cell viability was not affected after exposure to RA-RF. As shown in Figure 1B, the A549 cells were pre-treated with RA-RF (0–200 µg/mL) and followed by a non-toxic dose of PMFF (200 µg/mL). Our results showed that the combination of RA-RF and PMFF significantly diminished cell viability in a dose-dependent manner. The 20% inhibitory concentration (IC_20_) of the co-treated mixture was approximately 25 µg/mL and the 50% inhibitory concentration (IC_50_) was higher than 200 µg/mL.

In addition, the apoptosis assay was used to confirm the cause of the cell viability reduction by the combination. As shown in Figure 1C, the combination of RA-RF and PMFF had no effect on cells apoptosis and necrosis compared to the control. The non-toxic concentrations of RA-RF in the range of 0–20 µg/mL were used for all of the following experiments.

### 3.4. RA-RF Inhibits the Intracellular ROS Production

**Figure 2:**
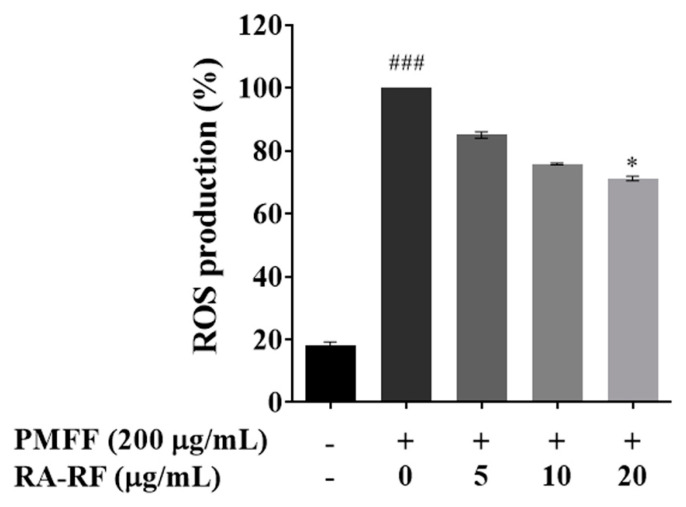
The intracellular ROS production in A549 cells exposed to PMFF (200 µg/mL) by the presence of RA-RF (0–20 µg/mL). The mean ± SEM are shown as ^###^ *p* < 0.001 vs. the control group; * *p* < 0.05 vs. PMFF group. The data were independently performed in triplicate.

PM reportedly induces the production of ROS in A549 cells [35,36]. The effects of RA-RF on intracellular ROS production in PMFF-treated A549 cells were determined by DCFH-DA assay. As shown in Figure 2, our result demonstrated that PMFF drastically increased the level of ROS production approximately 5-fold (*p* < 0.001) compared to the control, whereas 20 µg/mL of RA-RF significantly decreased the percentage of ROS formation (*p* < 0.05). This result strongly suggested that RA-RF could inhibit intracellular ROS induction, which typically causes cellular and oxidative damage in PMFF-induced A549 cells.

### 3.5. RA-RF Reducing the Inflammatory Cytokines and COX-2 Transcript

**Figure 3:**
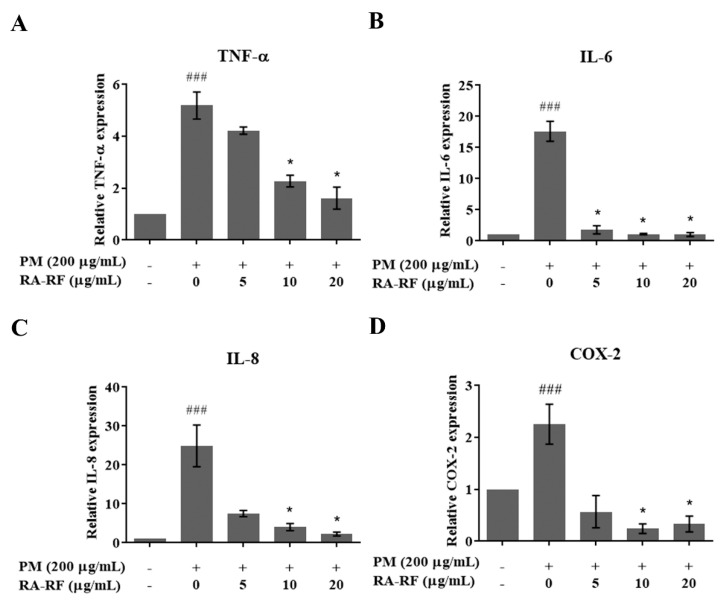
The gene expression profiles of inflammatory cytokines including TNF-α (**A**), IL-6 (**B**), IL-8 (**C**), and COX-2 (**D**) in A549 cells treated with PMFF (200 µg/mL) in the presence of RA-RF (5–20 µg/mL). The mean ± SEM are shown as ^###^ *p* < 0.001 vs. the control group; * *p* < 0.05 vs. PMFF group. The data were independently performed in triplicate.

PM induces oxidative stress, triggering inflammatory cytokines such as IL-6, IL-8, TNF-α, and COX2 enzyme [11,37]. As shown in Figure 3A–D, PMFF considerably induced the mRNA expression of TNF-α, IL-6, IL-8, and COX2, whereas RA-RF-treated cells showed significantly decreased expression of those transcripts. This result revealed that RA-RF could reduce the regulation of inflammatory cytokines and COX2 enzyme induced by PMFF.

### 3.6. RA-RF Inhibits Invasion, Migration and MMP-9 Activity of A549 Cells Induced by PMFF

**Figure 4:**
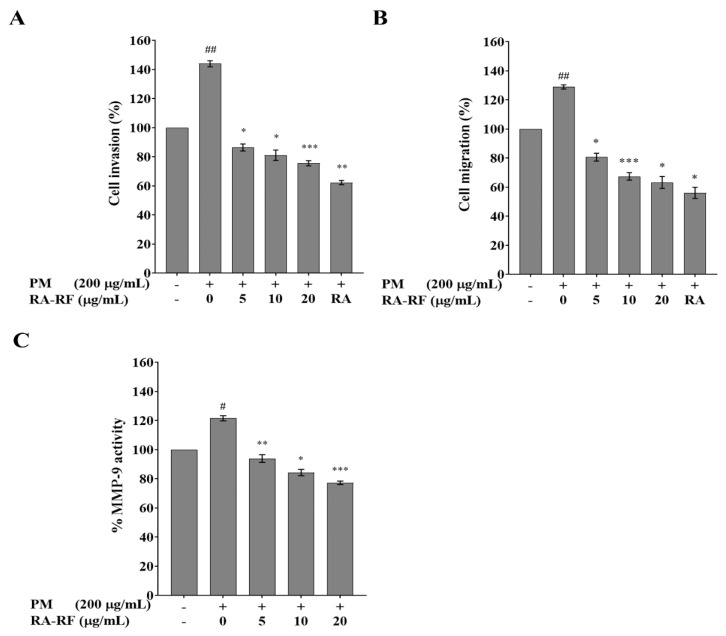
Inhibitory effects of RA-RF on invasion (**A**), migration (**B**), and MMP-9 activity (**C**) of PMFF-treated A549 cells. The mean ± SEM are shown as ^#^ *p* < 0.05, ^##^ *p* < 0.01 vs. the control group; * *p* < 0.05, ** *p* < 0.01, *** *p* < 0.001 vs. PMFF group. The data were independently performed in triplicate.

PM plays an important role in lung cancer formation and metastasis [13]. In this study, the anti-metastasis of RA-RF on PM-treated cells was investigated using Matrigel invasion and Transwell migration assays. As shown in Figure 4A, the invasive cells with PMFF treatment were increased by 1.44-fold (*p* < 0.01) compared to the non-treated cells. This revealed that treatment with 5, 10, and 20 μg/mL RA-RF significantly inhibited the invasion of PMFF-treated cells in a dose-dependent manner compared with commercial RA (5 μg/mL). Meanwhile, PMFF-treated cell migration significantly increased to 1.29-fold (*p* < 0.01); conversely, RA-RF (5–20 µg/mL) treatment significantly decreased PM-mediated migration in a concentration-dependent pattern compared to 5 μg/mL commercial RA (Figure 4B). Moreover, RA-RF (5–20 µg/mL) considerably reduced MMP-9 activity via a dose–response relationship (Figure 4C). In summary, polyphenols in RA-RF are capable of suppressing the invasion, migration, and MMP-9 activity of PMFF-induced A549 cells.

### 3.7. RA-RF Diminishes AP1, NF-κB and Akt Signaling Pathways

**Figure 5:**
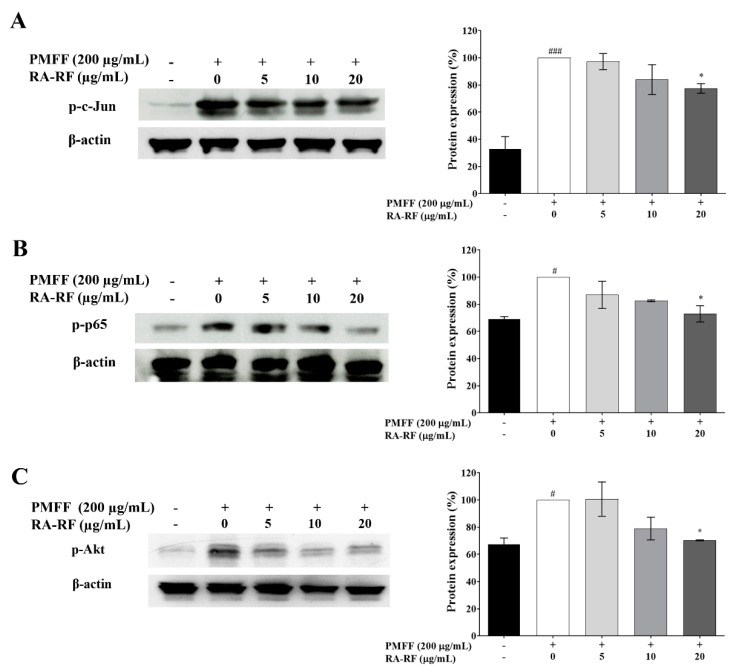
The protein expression of p-c-Jun (**A**), p-65-NF-κB (**B**), and p-Akt (**C**) in A549 cells exposed to PMFF (200 µg/mL) in the presence of RA-RF (5, 10, and 20 µg/mL) determined by Western blot analysis. Error bars indicate SD. The mean ± SEM are shown as ^#^ *p* <0.05, ^###^ *p* < 0.001 vs. the control group; * *p* < 0.05 vs. PMFF group. The data were independently performed in triplicate.

It has been reported the ROS-induced inflammation and metastasis are mediated through AP-1, NF-κB, and Akt signaling [38]. Therefore, this study determined the inducing effect of PMFF and the inhibitory effect of RA-RF on AP-1 (c-Jun), NF-κB (p-65), and Akt phosphorylation in A549 cells via Western blot analysis. As shown in Figure 5A–C, PMFF induced the phosphorylation of c-Jun, p-65-NF-κB, and Akt, whereas RA-RF (20 µg/mL) significantly inhibited the expression level of these signaling molecules in PMFF-treated cells. Collectively, RA-RF potentially inhibited the c-Jun, p-65-NF-κB, and Akt signaling pathways.

## 4. Discussion

Epidemiological studies have recently shown that PM is a crucial environmental contaminant related to various diseases such as pulmonary disease, cardiovascular disease, and lung cancer due to its ability to activate various inflammatory signaling pathways [39]. PM can also trigger both in vitro and in vivo determinations of the inflammatory response [3,40], metastasis induction [41], and other respiratory diseases [4]. Among several chemical components such as PAHs, metals, and trace elements, it was found that PAHs are predominantly absorbed in PM [39,42,43]. The PMFF samples were analyzed, and the concentrations of the 15 measured PAHs ranged from 0 to 2.094 ng/m^3^, with the highest component of PAHs being 2.094 ng/m^3^ of benzo[ghi]perylene, followed by, in order, indeno[1,2,3,-cd]pyrene, benzo[b]fluoranthene, and benzo[a]pyrene. Similarly, several studies in northern Thailand found that PM-bound PAHs were highly contaminated in the burning period [26,44,45,46,47].

These major PAH compounds were consistent with the data in our study. However, the PAH concentrations were lower than the values reported by Pooltawee et al. [45] in March 2014 in Phayao (nearly 180 ng/m^3^) and higher than those of the other studies in Chiangmai and Lamphun (0–1.98 ng/m^3^) [46,47]. This difference might be due to the period times, the area of PM collection, climate conditions such as season, temperature, rain, and humidity, and the scale of open burning derived from a number of hotspots [46,47].

To determine whether exposure to PAHs might cause significant effects in humans, health risk assessments can be calculated from the toxicity equivalent concentration (TEQ) based on PAH concentrations and toxic equivalent factors (TEFs) [26,48,49]. We found that the TEQ values in our study by using two equations as references from U.S. EPA (1993) [48] and Cecinato (1997) [49] were 1.37 ng/m^3^ and 1.69 ng/m^3^, respectively. These results were considerably higher than values reported by Wiriya W et al. and Yang N et al. [46,47]. In addition, the inhalation cancer risk (ICR) assessment was used for estimating the value of cancer risk from PAHs exposure by the equation ICR = TEQ × IUR_BaP_ [26,50] and IUR_BaP_ is the inhalation unit of risk defined as the risk of cancer from a lifetime (70 years) of inhalation of the unit mass of BaP, which is recommended as 8.7 × 10^−2^ m^3^/μg by the World Health Organization (WHO, 2000) [51]. The ICR values > 10^−4^ are potential cancer risk [26,52] and the values of our study were in the range of 1.19 × 10^−4^ and 1.47 × 10^−4^, which are similar to the previous report in Nan province, Thailand, in 2018 (1.01 × 10^−4^ and 1.33 × 10^−4^) [26], indicating potential cancer risk.

Based on the International Agency for Research on Cancer (IARC), PAHs are classified as carcinogenic, probably carcinogenic (class 1, 2A or 2B), and not classifiable (class 3) to humans [53]. Interestingly, our results detected all PAHs of high molecular weight, including benzo[a]pyrene (class 1), dibenz[a,h]anthracene (class 2A), benz[a]anthracene, benzo[b]fluoranthene, benzo[k]fluoranthene, chrysene, Indeno[1,2,3-cd]pyrene (class 2B), benzo[ghi]perylene, fluoranthene, and pyrene (class 3). The health effects of human inhalation exposure to PMFF containing PAHs from fire haze could induce oxidative stress, inflammation, invasion, migration, and risk of lung cancer [13,54,55,56]. Furthermore, these harmful effects might be also caused by metals, endotoxins, and other elements in PMFF [57,58]

In this investigation, PAH content in PMFF, cytotoxicity, ROS, pro-inflammatory cytokines, invasion and migration effects, and the molecular mechanism of RA-RF on A549 cells induced by PMFF were determined. In the cell viability test, we found that RA-RF (0–800 μg/mL) and PMFF (0–200 μg/mL) had no effect on cytotoxicity, but the high concentrations of PMFF (400 and 800 μg/mL) significantly decreased cell survival. The cytotoxic effects of PMFF may depend on the concentration of PAHs, metals, endotoxins, and other elements [57,58]. Interestingly, co-treated PMFF with RA-RF had no effect on cell apoptosis and necrosis. The cell death types of A549 exposed to PMFF may be autophagy and should be further investigated.

Oxidative stress is a consequence of the excessive ROS activity generated while antioxidant defenses are suppressed [59]. Numerous reports have demonstrated that oxidative stress plays an important role in the cytotoxicity, inflammatory response, and carcinogenesis of the PM-induced cells [58,60,61,62]. The studies have shown that PM exposure increases intracellular ROS generation in human umbilical vein vascular endothelial cells [63], human microvascular endothelial cells [64], and human adenocarcinoma A549 cells [65].

In the current study, we investigated the effect of RA-RF polyphenol on alleviating the production of ROS treated by PMFF in A549 cells. Our result illustrated that PMFF induced the level of ROS production, which is consistent with previous studies showing a significant increase in the ROS level in A549 cells after exposure to PM [66,67]. Importantly, non-cytotoxic doses of RA-RF significantly suppressed the oxidative stress in PMFF-induced A549 cells by diminishing ROS production. These findings are correlated with our previous reports that the extracts from perilla reduced oxidative stress [22,68]. Additionally, RA decreased the level of ROS in RAW macrophages and asthma mouse models [69,70]. Collectively, our results indicate that RA-RF can suppress intracellular ROS generation in PMFF-stimulated A549 cells, and thus may protect against oxidative stress and injury in lung epithelial cells induced by air pollution.

It has been reported that high PM content is directly associated with an upregulation in ROS and proinflammatory responses [10,11,12]. Our experiment showed that PMFF increased the mRNA expression of IL-6, IL-8, TNF-α, and COX-2 in the treated cells. Consistent with the results of previous studies, PM from wildfire and wood smoke emissions produced high levels of IL-6, IL-8, TNF-α, and COX-2 both in vitro and in vivo [71,72,73]. Our results also demonstrated that RA-RF, a natural polyphenol component found in perilla, reduced IL-6, IL-8, TNF-α, and COX-2 gene expression. This finding is in agreement with our previous report showing that RA extracted from perilla reduced mRNA levels of pro-inflammatory cytokines [20,22,74]. Additionally, RA-rich extract from *Trichodesma khasianum* leaves could diminish ROS production and inflammatory modulators such as IL-6, TNF-α, and COX-2 levels in vitro and in vivo [75]. Commercial RA also alleviated IL-6, IL-8, TNF-α, and COX-2 levels in both cell and animal models [76,77,78,79,80].

In addition to the stimulation of ROS production, ROS overproduction is a distinctive feature of cancer progression and resistance to medical therapy. PM plays a crucial role in ROS hyperactivation and cancer metastasis and previous studies have shown that A549 cell invasion and migration were enhanced by PM [13,81]. Similarly, our results demonstrated that PMFF significantly induced the invasion, migration, and MMP-9 activity of A549 cells. On the other hand, these metastatic processes were suppressed dose-dependently by RA-RF. Our previous study found that the perilla leaves extract, which mainly contains RA and other polyphenols, strongly inhibits invasion and migration via diminished MMP-9 secretion and activity in breast cancer cells [19]. This result is consistent with other studies showing that the RA suppressed cancer cell invasion, migration, and MMP activity/expression for colon carcinoma cells [82], pancreatic cancer cells [83], liver cancer cells [84], and human glioma cells [85].

Several studies have demonstrated PM-induced pulmonary inflammation via oxidative stress, autophagy, and cell apoptosis [86]. Moreover, PM-induced overproduction of ROS plays an important role in the inflammatory process and promotes lung cancer metastasis [6]. Therefore, controlling the oxidative stress may be a potential target for preventing or reducing PM-induced pulmonary inflammation and lung cancer metastasis.

The AP-1 is a ubiquitous dimeric protein complex of c-Jun and Fos. The expression level and phosphorylation in post-translation modifications regulate the activity of AP-1 [87]. The inhibition of AP-1 activity has been shown to reduce inflammation [88], invasion, and migration [30]. Several studies have demonstrated that the phosphorylation of c-Jun can be induced by PM in many cell types such as human dermal fibroblasts [41], human keratinocytes cells [89], RAW macrophage cells [90], hepatocellular carcinoma cells [91], and human bronchial epithelial cells [92].

Our results found that PMFF enhanced c-Jun phosphorylation, whereas RA-RF suppressed the enhancement in A549 cells treated with PMFF. This work resembles previous studies showing that the ethanolic extract of perilla leaves disrupted c-Jun phosphorylation [93]. Moreover, RA-rich extract from perilla seed meal could reduce RANKL-induced c-Jun translocation in RAW macrophage cells [74]. In addition, RA alone exerted protective effects against oxidative stress, inflammation, invasion, migration, and MMP-9 protein expression through the AP-1 signaling cascade [94,95].

NF-kB is an inducible transcription factor regulating a set of genes involved in inflammatory responses and cancer cell migration [96]. The classic NF-kB is composed of RelA (p65)/c-Rel heterodimers. This complex was phosphorylated and translocated to the nucleus for activating the expression of the target genes [97]. Recent studies show that the NF-κB signaling cascade can be activated via high production of ROS stimulating the inflammation and immune response, and this activation can also develop the migration ability of human lung carcinoma [98,99]. Additionally, NF-κB is also responsible for the inflammatory and metastatic effects of PM in both in vitro and in vivo [11,100,101,102]. Here, we found that PMFF drastically induced the phosphorylation of p65-NF-κB in A549 cells. This finding is consistent with several previous studies showing that PM increased the phosphorylation of p65-NF-κB [90]. In our study, RA-RF evidently suppressed the phosphorylation of p65-NF-κB in A549 cells treated with PMFF. Other reports showed that the perilla extract rich in RA [74] and RA compounds had a considerable inhibitory effect on inflammation via suppression of the NF-κB signaling pathway during both in vitro and in vivo experiments [103,104]. Moreover, RA inhibited the invasion and migration of human glioma cells [85] and human hepatoma cells through the PI3K/Akt/NF-κB signaling pathway [105].

Furthermore, it is well known that the PI3K/Akt signaling pathway plays an important role in cell inflammation, cell apoptosis, angiogenesis, and metastasis [106]. PM activated ROS production and the Akt signaling pathway, leading to inflammation of the human bronchial epithelial B2B cell line [107], invasion, and migration of hepatocellular carcinoma cells [108]. We have shown that PMFF augmented Akt phosphorylation but RA-RF suppressed the phosphorylation activity in PMFF-treated cells. Likewise, RA reduced the Akt signaling cascade, lowering TNF-α and IL-6 levels in rat models [79]. Moreover, RA suppressed the PDPK1/Akt/mTOR pathways via LPS-induced neuroinflammation both in vitro and in vivo [109]. Moreover, the invasion and migration of cancer cells were inhibited by RA through the Akt signaling cascade [110,111,112].

## 5. Conclusions

The inhibitory effects of RA-RF on oxidative stress, inflammation, and metastasis caused by PMFF induction in cancer cells have never been reported. Our report is the first to show that RA-RF from perilla seed meal exerted anti-oxidative stress, anti-inflammatory, and anti-metastasis effects on PMFF-induced A549 cells. It is concluded that RA-RF markedly suppressed the ROS production induced by PMFF, resulting in reduced IL-6, IL-8, TNF-α, and COX-2 mRNA expression and inhibited the metastasis cascade affecting MMP-9 activity, which reduced cancer cell invasion and migration via the AP1, NF-κB, and Akt signaling pathways. However, further in vivo study of RA-RF should be performed to explore its use as an inhalation agent for preventing lung inflammation and cancer metastasis induced by PM exposure.

## Figures and Tables

**Table 1:** List of the primer pairs in the present study.

Gene	Sequence
TNF-α	Forward: 5′-CCC AGG CAG TCA GAT CAT CTT C-3′
	Reverse: 5′-AGC TGC CCC TCA GCT TGA-3′
IL-6	Forward: 5′-ATG AAC TCC TTC TCC ACA AGC-3′
	Reverse: 5′-GTT TTC TGC CAG TGC CTC TTT G-3′
IL-8	Forward: 5′-AGA TAT TGC ACG GGA GAA-3′
	Reverse: 5′-GAA ATA AAG GAG AAA CCA-3′
COX-2	Forward: 5′-CCC TTG GGT GTC AAA GGT AA-3′
	Reverse: 5′-GCC CTC GCT TAT GAT CTG TC-3′
GAPDH	Forward: 5′-GAA GGT GAA GGT CGA GTC A-3′
	Reverse: 5′-GCT CCT GGA AGA TGG TGA T-3′

## Data Availability

Not applicable.
